# Wind-Driven Roof Turbines: A Novel Way to Improve Ventilation for TB Infection Control in Health Facilities

**DOI:** 10.1371/journal.pone.0029589

**Published:** 2012-01-09

**Authors:** Helen Cox, Rod Escombe, Cheryl McDermid, Yolanda Mtshemla, Tim Spelman, Virginia Azevedo, Leslie London

**Affiliations:** 1 Médecins Sans Frontières, Cape Town, South Africa; 2 Burnet Institute for Medical Research, Melbourne, Australia; 3 Department of Infectious Diseases and Immunity, Imperial College London, London, United Kingdom; 4 City of Cape Town Health Department, Cape Town, South Africa; 5 University of Cape Town, Cape Town, South Africa; National Institute for Infectious Diseases (L. Spallanzani), Italy

## Abstract

**Objective:**

Tuberculosis transmission in healthcare facilities contributes significantly to the TB epidemic, particularly in high HIV settings. Although improving ventilation may reduce transmission, there is a lack of evidence to support low-cost practical interventions. We assessed the efficacy of wind-driven roof turbines to achieve recommended ventilation rates, compared to current recommended practices for natural ventilation (opening windows), in primary care clinic rooms in Khayelitsha, South Africa.

**Methods:**

Room ventilation was assessed (CO_2_ gas tracer technique) in 4 rooms where roof turbines and air-intake grates were installed, across three scenarios: turbine, grate and window closed, only window open, and only turbine and grate open, with concurrent wind speed measurement. 332 measurements were conducted over 24 months.

**Findings:**

For all 4 rooms combined, median air changes per hour (ACH) increased with wind speed quartiles across all scenarios. Higher median ACH were recorded with open roof turbines and grates, compared to open windows across all wind speed quartiles. Ventilation with open turbine and grate exceeded WHO-recommended levels (60 Litres/second/patient) for 95% or more of measurements in 3 of the 4 rooms; 47% in the remaining room, where wind speeds were lower and a smaller diameter turbine was installed.

**Conclusion:**

High room ventilation rates, meeting recommended thresholds, may be achieved using wind-driven roof turbines and grates, even at low wind speeds. Roof turbines and air-intake grates are not easily closed by staff, allowing continued ventilation through colder periods. This simple, low-cost technology represents an important addition to our tools for TB infection control.

## Introduction

Tuberculosis notifications in South Africa have increased fivefold over the last two decades, with 1% of the population currently estimated to develop active TB each year [1]. These data suggest that TB control has failed in South Africa, despite efforts aimed at increasing case detection and improving outcomes [2]. Clearly the high adult HIV prevalence of 18% in South Africa has driven the TB case load dramatically, with consequent increased burdens on all levels of the health system [3]. In this context, one area that has received insufficient attention is the issue of TB infection control in health facilities. Some impetus for TB infection control has arisen following the emergence of drug-resistant strains of TB; multidrug resistant- and extensively drug resistant-TB, for which evidence of nosocomial transmission to both patients and health care workers exist [4], [5]. Given high rates of infectious active TB disease (both drug-susceptible and drug-resistant) and high proportions of vulnerable HIV-infected individuals, over-burdened health facilities are likely to be extremely high risk areas for TB transmission.

TB infection control interventions are categorized as administrative, environmental and personal protective [6]. Activities under these categories should be implemented in a package to reduce TB transmission risk. Environmental controls aim to reduce the concentration of infectious droplet nuclei in air, and to control the direction of contaminated airflow. Increasing room ventilation is the mainstay of environmental controls. Ventilation may be natural, by simply opening windows and/or doors, or mechanical, through the use of air extraction and/or injection fans. Natural ventilation has been identified as an effective measure to control health care infections by WHO since 2007 [7], which currently recommends that natural ventilation in general wards and outpatient departments should be no less than 60 litres/second/patient [8]. The US Centers for Disease Control have used a different ventilation measure, and recommended at least 6, and preferably 12 air changes per hour (ACH) for rooms in which airborne droplet nuclei are present [9]. There is however, limited evidence with which to definitively recommend a ventilation rate that will reduce the TB transmission risk to an acceptable minimum.

South Africa has more than 6,500 primary health care facilities, attended by patients who may be clinically or sub-clinically infected with HIV and/or TB. Powered mechanical ventilation is expensive to install and maintain, and is therefore neither feasible nor practical across these facilities. Many facilities lack consistent electricity supply and skilled technicians to install and maintain ventilation systems are scarce. Increasing ventilation using simple technology driven by natural forces may provide a promising alternative. One such measure is the installation of wind-driven roof turbines, ducted through ceilings, in order to increase the extraction of potentially contaminated air. These devices are currently in routine use around the world, primarily for ventilating roof spaces and industrial buildings. Wind-driven roof turbines are a low-cost, low-maintenance technology, not requiring electricity. As devices to assist in improving airflow and ventilation, this technology could potentially contribute to control of TB transmission. Given the need to develop an evidence-base for practical interventions to reduce TB transmission risk in health facilities, we aimed to assess the efficacy of wind-driven roof turbines to achieve recommended ventilation rates, compared to current recommended practices for natural ventilation (opening windows), in primary care clinic rooms in Khayelitsha, South Africa. Khayelitsha, home to approximately half a million people, is an urban township with extremely high burdens of TB, both drug-susceptible and drug-resistant, and HIV infection [10], [11].

## Methods

### Intervention

**Figure 1:**
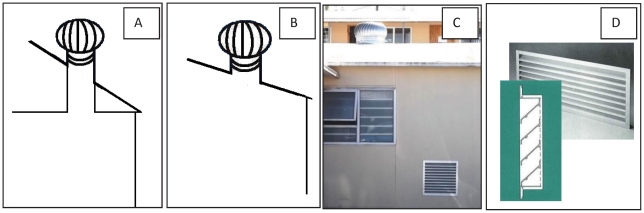
Installation of roof turbines and air-intake grates. A: Roof turbine with ducting from roofline to ceiling, B: roof turbine without ducting (no roof space), C: photograph of outside of clinic room, showing roof turbine and air-intake grate, and D: design of louvred air-intake grates used (effective opening area is 0.68 of actual grate area).

Wind-driven roof turbines were installed in rooms in three primary care clinics in Khayelitsha in 2008/09. Roof turbines were ducted into rooms, either directly in cases where there was no roof space, or via a duct from the roof to the level of the ceiling (figure 1). Flexible, ridged ducting of 500 mm diameter was used to duct turbines (regardless of turbine size). All roof turbines installed were constructed of aluminium, spherical in shape and locally supplied (Bigbird roof turbine ventilators, Modern Products, www.modernproducts.co.za). In all cases, air-intake into the room was provided through a louvred air-intake grate, mounted in walls or doors, depending on where the freshest air could be provided. The cost of the material and installation of the roof turbine and air-intake grates ranged from 200–350 USD per turbine.

Four clinic rooms were chosen for measurements, on the basis of representing different building types, roof designs and room layouts (Table 1). Taking account of room volume and assuming one patient per room, the WHO-recommended ventilation rate of 60 L/second/patients can be converted into a target ACH for each room; these values ranged between 6.5 ACH for the largest room (room 4) to 10.0 ACH for the smallest (room 1) (Table 1).

**Table 1:** Description of clinic rooms, roof turbines and air intake grates.

	Room 1	Room 2	Room 3	Room 4
Clinic	A	B	C	C
Room function	Counselling room: individual counselling sessions for HIV and TB treatment (one patient, one counsellor)	TB room: Diagnosed TB patients receive their daily medication (one patient, one nurse)	Clinical consulting room: Medical consultation (one patient, one doctor)	TB suspect room: Patients with TB symptoms are assessed and instructed to give sputum (one patient, one nurse)
Building structure	Brick, gabled roof (purpose built)	Brick, gabled roof (converted house)	Pre-fabricated building (dry wall, no roof space)	Pre-fabricated building (dry wall, no roof space)
Room volume	21.7 m^3^	32.1 m^3^	29.2 m^3^	33.0 m^3^
WHO recommended ventilation rate*	10.0 ACH	6.7 ACH	7.4 ACH	6.5 ACH
Roof turbine; External diameter, Installation	300 mm, ducted between roofline and ceiling	500 mm, ducted between roofline and ceiling	500 mm, no ducting, ceiling attached to roof line	500 mm, no ducting, ceiling attached to roof line
Air-intake grate; Position, Grate area, Effective open area	Door, 1.6 m^2^, 1.1 m^2^	External door, 2.4 m^2^, 1.6 m^2^	External wall, 1.1 m^2^, 0.69 m^2^	External wall, 2.3 m^2^, 1.6 m^2^

*Calculated based on recommendation of 60 L/second/patient using room volume and assuming one patient per room.

### Experimental design

Room ventilation was measured over 5–8 minute periods using a carbon dioxide gas tracer technique, where CO_2_ is released into a room, mixed thoroughly with room air, and concentration-decay recorded over time [12], [13]. CO_2_ was measured using an infra-red gas detector (OrionPlus, MSA, Germany), reading at 15 second intervals. The gas probe was placed in the centre of the room at head height for a seated person. Wind speed was measured simultaneously using a data-logging anemometer (Inspeed, USA); one-minute averages were then averaged over the measurement period and results presented as mean wind speed. The anemometer was positioned on the roof at the same level as the roof turbine and no more than 2 metres from the roof turbine.

An airflow capture hood (Lo-Flo Balometer, Alnor, USA) was used to measure flow directly through the roof turbine during the experiment. The capture hood was used to take manual spot measurements at 1 minute intervals during each measurement period, with the results averaged over the measurement period. These measurements were used to calculate the contribution to total room ACH from extraction through the roof turbine directly. Internal room temperature and external air temperature were measured at the start and end of each experimental session. Measurements were taken across three scenarios:

1. All doors and windows closed (turbine and grate blocked),
2. One window opened (door closed and turbine and grate blocked) and
3. Only roof turbine and air-intake grate open (windows and doors closed).

All 3 scenarios were tested in each experiment (not necessarily in order), with a maximum of 3 experiments per day. Experiments were conducted across the year under different weather conditions during normal clinic open hours (8 am through 5 pm), although rooms were not in use during experiments. During measurements, one to two persons were in each room to conduct the experiment.

### Data analysis

All manual data were entered on to standard recording forms and later on to a computerised database (Microsoft Excel 2007). Data on declining CO_2_ concentration decay were downloaded from the gas analyser and transferred to Excel for analysis of the slope of the graph of the natural logarithm of CO_2_ concentration against time (used to determine ACH). Data were later analysed with PASW Statistics (version 18, 2009, SPSS, USA). As wind speed and ACH were significantly skewed, median values were used to describe wind speed and ACH. Medians were compared between groups with the Mann-Whitney test and the Kruskal-Wallis test for multiple groups. Proportions were compared between groups using the Chi-squared test. Median (quantile) regression was used to assess the magnitude of the association between room ventilation and wind speed. All reported p-values are two-tailed and for each analysis, p<0.05 was considered significant.

## Results

Between April 2009 and March 2011, 332 separate room ventilation measurements were taken on 46 days from the four rooms (3 clinics) and across the three scenarios tested. Measurements in room 2 were stopped in November 2009 due to construction work at the clinic which changed the room design. Median and range of wind speeds are given in table 2. Overall, median wind speed recorded during ventilation measurements was 10.1 km/hr (range 1.0–50.0 km/hr). Clinic A sits in a geographical depression in Khayelitsha and recorded consistently lower wind speeds. External air temperatures for each ACH measurement ranged between 15.1°C to 30.4°C. Only small differences were observed between internal and external temperatures measured through experiments (mean difference = 0.4°C, standard deviation = 1.7°C).

**Table 2:** Distribution of measured wind speed (km/hr) by clinic.

Clinic	No. measurements	Median wind speed	Inter-quartile range	Range
**A**	156	7.8	4.8	1.2–18.7
**B**	19	11.3	5.9	7.1–18.4
**C**	157	11.9	7.1	1.0–50.0
**Total**	332	10.1	6.5	1.0–50.0

**Figure 2:**
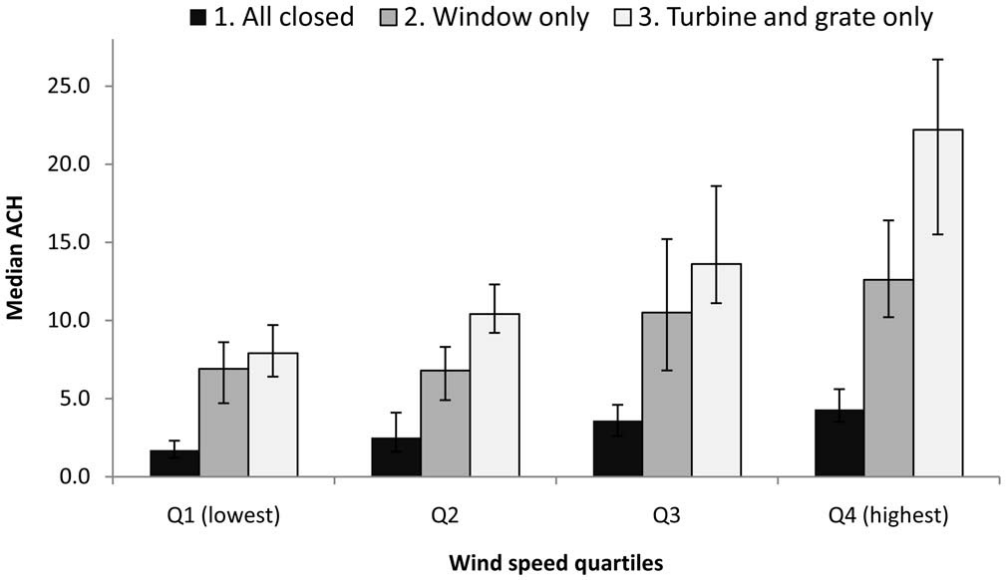
Median ACH by wind speed quartiles (Q) and for each scenario, for all rooms combined, given for each of the 3 scenarios tested (error bars are 95% confidence intervals for the median).

Pooled data from all rooms and ventilation scenarios were used to create quartiles of wind speed. Median ACH for each of the three scenarios (windows, turbine and grate closed; only window open, turbine and grate open with window closed), was calculated according to wind speed quartile (figure 2). Median ACH increased significantly across wind speed quartiles for each of the three scenarios (p<0.000).

For each room, median and range of measured ACH, along with the percentage of ACH measurements that were above the equivalent ACH based on WHO-recommended ventilation rates are shown in table 3 for scenarios 2 and 3 (window only open, and turbine and grate open). Roof turbines produced higher median ACH measurements and greater proportions of measurements above the WHO recommendation than open windows alone. These differences were statistically significant for rooms 2, 3 and for all rooms combined. Ventilation was below recommended in room 1 for 70% of measurements with just the window open and approximately half of measurements with the turbine and grate. In room 4, both median ACH and the proportion of measurements above recommended were high for both the window and turbine scenarios. Overall, three of the four rooms were above WHO recommended levels of ventilation for at least 95% of all measurements with the roof turbine and grate.

**Table 3:** Median ACH, range and number of measurements that are above the WHO recommendation for each room, comparing window only and turbine and grate only.

	Room 1	Room 2	Room 3	Room 4	Total(all rooms)
***WHO recommended ACH***	***10.0***	***6.7***	***7.4***	***6.5***	
**Window only (scenario 2)**					
Number of experiments	51	5	26	24	106
Median ACH	8.1	3.7	7.3	11.4	8.5
IQR	6.2–10.1	2.7–4.8	3.5–11.2	7.2–15.7	5.5–11.5
Range	2.1–29.6	2.0–4.6	1.3–16.4	2.4–24.8	1.3–29.6
No. above WHO recommendation	15 (29%)	0 (0%)	13 (50%)	20 (83%)	48 (45%)
**Turbine and grate only (scenario 3)**					
Number of experiments	43	7	23	22	95
median ACH	8.9	13.5	16.7	13.4	11.3
IQR	5.8–12.0	10.6–16.4	9.4–24.1	8.0–18.9	7.2–15.4
Range	3.2–21.6	9.2–17.2	8.4–30.0	6.3–37.7	3.2–37.7
No. above WHO recommendation	20 (47%)	7 (100%)	23 (100%)	21 (95%)	71 (75%)
**Comparison between scenarios 2 and 3**					
P value (comparison of medians)	0.28	0.004	<0.000	0.07	<0.000
P value (comparison of proportion above WHO recommendation)	0.09	0.004	<0.000	0.40	<0.000
% Contribution of roof turbine to ACH* (95% CI)	71 (64–78)	61 (48–75)	49 (43–55)	40 (36–43)	58 (53–62)

*Calculated by using the measurement of actual flow through the turbine, at the same time as overall ACH measurement.

For rooms 1 to 4 respectively, mean ACH attributable to flow through the turbine were 6.9, 7.8, 8.1 and 6.3. Pair-wise, these values equate to between 40% and 71% of air changes in each room, and 58% (95% CI 53–62) of total ACH for all rooms combined (table 3).

**Figure 3:**
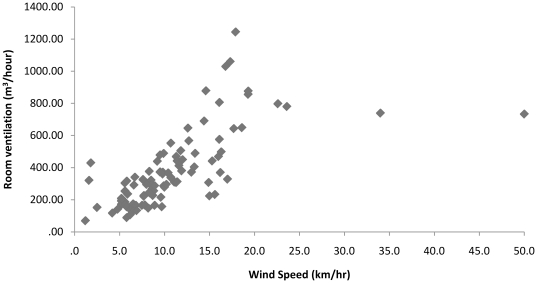
Association between wind speed and room ventilation (m^3^/hour) for measurements with the roof turbine and air intake grate, all rooms combined.

There appeared to be a significant correlation between wind speed and room ventilation for all rooms combined using the data from scenario 3 with the roof turbine and air-intake grate open (Spearman correlation coefficient = 0.78, p<0.000) (figure 3). However, at higher wind speeds (above 20–25 km/hr) this association appeared to fall away. Using a median (quantile) regression where outcome is room ventilation and the predictor is wind speed, an increase in wind speed of 1 km/hr is statistically significantly associated with a 22.8 m^3^/hour increase in room ventilation (p<0.001).

## Discussion

This study has shown that high rates of room ventilation, recommended for the prevention of airborne TB transmission, may be achieved using wind-driven roof turbines. Adequate ventilation rates were obtained across seasons, even with low wind speeds. This simple, low-cost technology therefore represents an important addition to our tools for TB infection control. Roof turbines are low-cost, require little maintenance and do not use electricity. As a result they are an attractive intervention for airborne infection control in low resource settings where the burden of TB is highest. Roof turbines, combined with grates, also have the benefit of being less dependent on behavioural adherence by staff; they are not easily closed by staff or patients, and thus allow continued ventilation through colder periods.

While a limited number of rooms were included in this study, the results suggest some guidance as to how roof turbines should be installed. Roof turbines appeared to work better in rooms 3 and 4; these are rooms in a clinic where there is no roof space and therefore no need to duct turbines between the roof line and the ceiling. In rooms 1 and 2, performance of the turbine was less effective. This may be the result of the ridged material used to duct turbines causing increased turbulence and airflow impedance, thereby decreasing the capacity of the turbine to extract air. Ducting material with a smooth surface is likely to perform better, but is more difficult to install. Ventilation was insufficient in room 1 using the roof turbine as installed. In addition to the duct through the ceiling, room 1 had a smaller sized turbine, and was located in the clinic with the lowest measured median wind speeds. These observations suggest that the larger turbines are more effective for the relatively small rooms assessed here and that if the turbine needs to be ducted, perhaps a larger turbine may be required to compensate for the adverse effect of ducting. However, further research is needed to inform more accurate recommendations on how turbines should be installed, including aspects of turbine size, turbine design, ducting material and appropriate ratio of size of air-intake grate to turbine size.

Cape Town is generally a windy city. Khayelitsha is located just 10 km from the Cape Town international airport, which reports average monthly wind speeds between 16 and 26 km/hr (9–14 knots) between the hours of 7 am and 7 pm [14]. Therefore, Khayelitsha is optimally placed to utilize wind driven mechanisms to increase ventilation, which may not be the case in many locations where health care facilities with high TB burden are found. However, for the size of roof turbines used here (300–500 mm external diameter), it appears that the capacity of the turbine to extract air may be surpassed at wind speeds greater than 20–25 km/hr, with no further increases in ventilation demonstrated above this.

An important consideration in natural ventilation is comfort for both staff and patients, especially in cooler seasons or climates. Although not systematic, discussions with staff who use the rooms included in this study suggest that the turbine and grate combination produces less direct drafts than does opening a window, and that they are able to warm rooms sufficiently in colder months using electric heaters. However, tolerance of cold air is likely to be higher in outpatient settings, such as in this study, compared to inpatient settings where patients are often extremely unwell and immobile. Hence, the use of turbines may not be appropriate in all settings and climates, particularly where outside temperatures often fall below zero degrees Celsius. The advantage of turbines over windows in this study is likely related to variability of wind direction. As roof turbines are driven by wind, regardless of direction, particularly when positioned above the top most point of the roof line, they are less subject to changing wind direction than windows with a fixed orientation.

Based on the measurement of airflow through the roof turbine, approximately 60% of measured ACH achieved with the roof turbine scenario was due to direct flow through the turbine. However, this measurement was highly variable across the rooms assessed, being highest in room 1, the newest brick building and lowest in rooms 3 and 4 in the prefabricated building. This variation and overall low figure most likely reflects the leakiness of these buildings, and the significant extent of airflow moving through gaps around doors and windows, particularly in older buildings. This is reflected in the increasing ACH measured with increasing wind speed, up to 4.3 ACH, even when all doors and windows are closed, when theoretically there should be no ventilation at all. Furthermore, it is possible that the resistance caused by placing the capture hood beneath the roof turbine duct during measurement impeded the airflow, and thus resulted in falsely low readings. These data highlight the advantages of measuring total room ACH using a gas tracer technique, rather than airflow through individual openings in rooms.

The use of the carbon dioxide technique allows an accurate assessment of ACH in real rooms, where there are multiple sites for air movement, both in and out of the room. The technique relies on an adequate mixing of gas through the room and assumes that gas decay as measured at a single point reflects that throughout the room. Given that the rooms included in this study are relatively small at approximately 30 m^3^, we feel that this is a valid assumption. For the sake of simplicity we compared three scenarios; firstly, when all windows and doors are closed, as is often the case in winter in health facilities, only a window opened as would be the case in summer during a consultation, and only the roof turbine and grate open. Clearly, there are many different combinations that could be tested, but the aim was to first determine the efficacy of roof turbines to reach WHO-recommended levels of ventilation and secondly to compare with the common scenario of opening a window. It is highly likely that in summer months when windows are often opened in addition to the turbine and grate, increased ACH will be created. Therefore, the use of roof turbines should not negate the simple message to open windows wherever possible.

Measurements of room ventilation using an open window were considerably lower in this study compared to previous assessments of natural ventilation [13]. This is most likely to result from the lack of cross ventilation in these relatively small clinic rooms, with only one window per room (a common design for small consulting rooms). Additionally, window designs which restrict the degree to which windows can be opened may also restrict airflow. Most windows in this study could only be partially opened. Cross ventilation, with adequate window openings, is one of the main drivers of natural ventilation. Indeed, based on previous studies [13], large windows and cross ventilation is likely to provide much greater air exchange than just a roof turbine (and grate) alone.

To date, the roof turbines and air-intake grates installed in Khayelitsha have required little maintenance and remain functioning as at installation (1.5–3 years). One of the earliest turbines, and some of the air-intake grates, in this study were found to leak in heavy rain. The roof turbine was appropriately re-installed by the supplier and the air-intake grate was raised higher above the ground to avoid heavy rain splashing up through the grate. We are therefore confident that the use of roof turbines remains a sustainable intervention.

This study suggests that roof turbines, installed in conjunction with air-intake grates have the capacity to significantly improve ventilation in health facilities. Although there is a lack of biological proof to definitively demonstrate the impact of ventilation on TB transmission, earlier studies utilising mathematical models strongly suggest that transmission can be reduced with increasing supply of fresh air [15], [16]. Thus improving ventilation, particularly in conjunction with other interventions, may help to reduce the risk of airborne infection. Natural ventilation can undoubtedly produce high rates of air exchange when there is cross ventilation and windows are large [13]. However, maintaining open windows often requires behavioural adherence, and cross ventilation may be absent, as in the rooms here. Thus many existing health facilities may benefit from the installation of roof turbines, at reasonable cost, in conjunction with current recommendations for open windows. We conclude that wind-driven roof turbines should be part of a range of tools available for improving TB infection control, particularly in low resource settings.
